# Urban gardening education: User reflections on mobile application designs

**DOI:** 10.1371/journal.pone.0310357

**Published:** 2024-09-12

**Authors:** Ewa Duda

**Affiliations:** Institute of Education, Maria Grzegorzewska University, Warsaw, Poland; Canakkale Onsekiz Mart University, TÜRKIYE

## Abstract

Mobile gardening applications offer a wide range of opportunities to shape the environmental behaviour of city dwellers, while stimulating action for greater access and contact with nature. Despite this, their educational potential is not sufficiently recognised and exploited. The aim of this qualitative research is to gain an in-depth understanding of the extent to which existing mobile apps can facilitate digital education for the development of green cities. For this purpose, the user insight approach has been applied. The study analyses 7 980 reviews of fourteen apps applications from Google Play Store. The results reveal the motivations behind users’ decision to download urban gardening apps and the features that facilitate or hinder their use. The obtained results are relevant not only for green information systems research but also for app developers, and those involved in the urban education process: city authorities, urban educators, pro-environmental associations, and grassroots activists, among others.

## Introduction

As urban populations continue to grow and housing demand increases, scarce urban green spaces are being replaced by apartment buildings [1], reducing people’s access to nature. The increasing presence of concrete and covering the streets with pavements is supposed to make it easier to maintain public spaces [2]. Urban air quality is poor due to heavy public transport [3] or the presence of smog, evidenced by the situation in New York during the wildfires in Canada [4]. Consequently, the right to breathe clean air is limited. Stressful, hectic lifestyles, and inadequate diets result in an increase of the number of people suffering from depression or other mental health problems [5].

The aforementioned environmental related issues can be alleviated by increasing the right to contact with nature through the development of so called “green cities”—urban spaces which „offers clean air and water, as well as pleasant streets and parks. Green cities are immune to natural disasters, and the risk of major infectious disease outbreaks in such cities is low” [6:4]. Urban cultivation of vegetables, fruits or herbs should be recognised as one of the key methods of developing green cities and, at the same time, offers the possibility of providing residents with a higher quality of a variety of food. Private urban gardening or small-scale urban agriculture [7, 8] takes place in residential lots, private or community gardens, allotment gardens, public space, or on the balconies of apartment buildings [9].

Undoubtedly, urban gardening (or urban agriculture) has many advantages in terms of sustainability [10]. It allows creation of enclaves of green urban spaces, even in small areas and including places that are difficult to access. Creation of gardens and even aquaponic systems on the roofs of buildings, in staircases, and in building walls has become more and more popular [11]. According to [12], the three main motivations for urban gardening, such as restoration, socialisation and food production, are not context-dependent and represent a source of well-being for urban dwellers regardless of gender, age, education level and income. Shared community gardening and agriculture strengthens interpersonal relationships and a sense of community belonging can develop as it builds social trust, capital, and attachment to nature. Urban gardening and agriculture are also significant for promotion of environmental education, intergenerational sharing of knowledge, and the growth of the eco-pedagogical nature of cities [13]. [14] suggest that community gardens supporting food self-production are beneficial to personal well-being as well as mental and physical health of those involved in gardening activities, particularly of those at risk of social exclusion. Given the benefits of urban gardening, it is worth supporting this process.

The widespread use of smartphones and mobile applications changes our way of functioning in the modern world. The number of active users of different applications increases each year. This trend was particularly reinforced during the pandemic [15]. That is why an effort should be made to solve the problems of today’s cities by using tools that are available to their users. Therefore, the motivation for the present research has been to explore the extent to which available mobile applications facilitate urban residents’ food self-provisioning (growing of one’s own food, especially fruits and vegetables). In particular, how a new way of learning, such as digital learning, can support the green transformation of cities. This topic may give rise to an interesting discussion, particularly when the need for sustainability of environment and food production is a key subject of the debate around climate change. The main research question (MRQ) of the study is:

MRQ: What opportunities are identified by users of mobile applications for urban gardening in applying digital learning in green urban transformation?

The paper is structured as follows: the following section is dedicated to literature regarding mobile applications, and is followed by a presentation of the research methodology and a description with an analysis of the empirical results, summarised by the studies’ conclusions.

## Related works

### Environmental mobile applications (green apps)

The growing concern for environmental issues and the rapid development of information systems (IS) have led to new technologies being seen as tools for educating people about significant societal challenges, including increasing pro-environmental knowledge and awareness. With the ever-increasing number of smartphone and other mobile device users, mobile applications have the potential to reach a broad audience [16]. The use of mobile applications in increasing ecological knowledge and shaping pro-environmental behaviour has gained favour in many areas. A popular approach involves using a game mechanism to enhance participants’ educational engagement. [17] proposed an application incorporating gamification elements to influence lifestyle habits for reducing CO_2_ emissions by individual families, such as encouraging them to turn down air conditioning and to use public transport rather than private vehicles etc. Their research reveals that adopting the “EcoIsland” application led to a change in user awareness. However, the motivation to reduce emissions was driven more by the desire to collect incentives and compete with family members rather than environmental concerns.

In turn, the developers of the “WasteApp” have identified the potential for mobile applications in supporting recycling habits [18]. They observed a positive impact on functioning benefits due to interaction within the application, as the users felt safe using its functionalities. Moreover, the application did not require an important level of emotional involvement to achieve its goals.

The developers of the RANAS mobile app also aided the approach for recycling behavioural changes applied in Singapore and came to similar conclusions [19]. Their findings showed that by incorporating the approach of tracking user behaviour and sense of ownership resulted in an increase in recycling behaviours by more than 20% and a reduction in contamination levels to less than 2%. According to the authors, the mobile application supports pro-environment behavioural changes and is a good training tool to educate people about recycling practices. [20] reviewed thirty-two applications for sustainable consumption. They found that the applications that show sustainability rankings to customers encouraged companies to take further care about the quality of their products as well as following good practices. The authors suggest that there is a need for further research to develop transparent communication of technical and specialist information that may not be understood by a common user, despite the fact that they are able to use the application. It is unclear whether the educational role of applications produces a rise of consumer awareness, or rather it discourages users by promoting complicated environment sustainability related indicators. The authors also note that the potential of applications to increase consumer awareness of natural resource management is not studied enough nor used, especially by conservation scientists and educators.

The articles exploring environmental mobile applications (green apps), include the texts focused on urban gardening. [21] introduce the PHYTO application for houseplant care. The automation process of horticulture covers the monitoring of the condition of plants such as: sensors controlling the quality of the environment allowing provision of adequate growing conditions, optimal hydration, adjustment of air temperature and humidity, monitoring of soil moisture and of optimal lighting. [22] also demonstrates the benefits of a home gardening application. The authors claim that the application serves as a platform to connect food self-provisioning (gardening) enthusiasts, as a sense of belonging to a community is one of the crucial factors influencing active engagement in the process. The ability to share information by people involved in gardening activities was cited as being extremely helpful for gardening, as well as being beneficial for the well-being of the community created by this application service. The opportunity to share their achievements in gardening has played a significant role.

### Present research

Analysing user opinions on software and applications provides valuable scientific insights, particularly for those designing effective learning tools [23]. One of the research methods used is sentiment analysis, which involves using “natural language processing, text analysis and computational linguistics to identify and extract subjective information in source materials. Sentiment analysis aims at determining the attitude of a speaker or a writer with respect to some topic or the overall contextual polarity of a document” [24:342]. However, due to the exploratory nature of the research, manual analysis is often employed. [25]. Despite numerous studies on user opinions, there is still little focus on applications supporting urban agriculture; an example is the study conducted by [26], where the authors analysed 1,522 reviews from Bangladeshi agricultural applications, exploring the issue of basic human values.

To address the identified research gap and deepen understanding in this area, the present study focuses on analysing user reviews of mobile applications for urban gardening education, which are scarcely represented in the literature. In addition to [22], who conducted qualitative research among users of a mobile gardening application, the study investigates the issues identified by the users that suggest opportunities to employ digital learning for green urban transformation. In particular, the study explores users’ motivations for using urban gardening applications and the types of users. The author’s intention is to gain an in-depth understanding of which app functionalities are most likely to be used, what barriers hinder the application use or discourage from using the app, and how these factors relate to the decision to download an application and, more importantly, to actively interact with it. It is essential to reach people who can create a solution for them [27]. As people spend a significant amount of time on their mobile phones, an educational application that enhances gardening skills would be particularly beneficial, especially in an area often overlooked. With the rapid population growth and rising living costs, the issue of self-gardening deserves a deeper exploration. To investigate these problems, the study is guided by the following detailed research questions (DRQ), which support the main study objective:

DRQ1: What factors influence users’ decisions to download urban gardening applications?

DRQ2: What features of urban gardening applications facilitate user learning?

DRQ3: What are the barriers that impede the use of urban gardening applications, or act as a deterrent to their adoption?

Understanding these issues may allow a better design of a mobile application for urban gardening as a digital learning tool to support the dwellers’ green urban transformation process and support their informal lifelong learning process.

## Methodology

**Fig 1:**
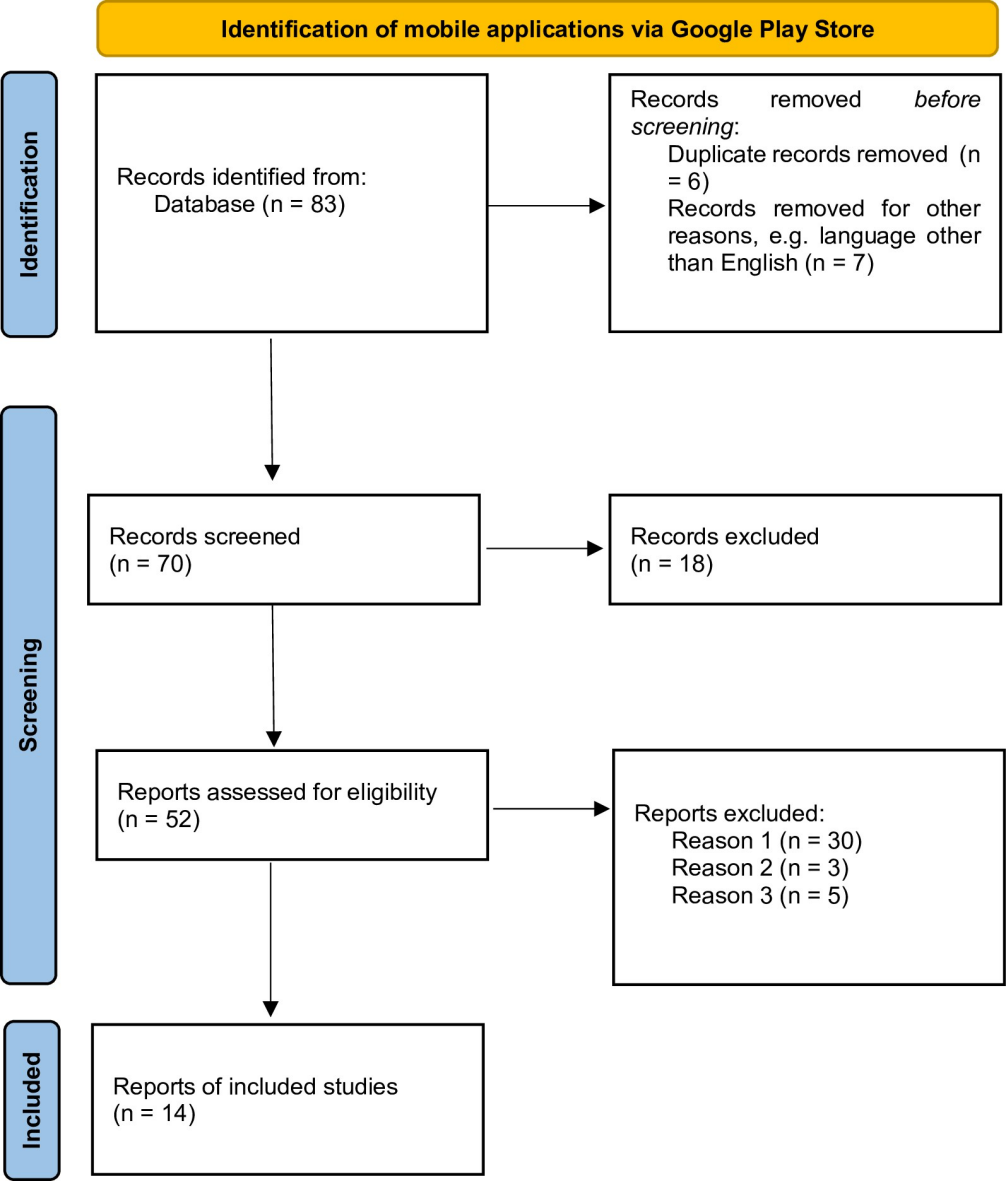
PRISMA 2020 flow diagram. Author’s own elaboration based on [28].

The method employed in the presented study is qualitative data analysis. The research material included opinions of mobile urban gardening application users. The study followed the Preferred Reporting Items for Systematic Reviews and Meta-Analyses (PRISMA) approach [28]. The PRISMA 2020 flow diagram along with the subsequent stages is presented in Fig 1.

### Procedure

Mobile applications were explored via the Google Play Store (GPS) platform. The first stage (Identification) covered a search applying the following criteria: English language versions, downloadable applications with user reviews published and available for public use. The keywords used were: "garden" and "farming". The next research step consisted of applying the snowball method. The purpose of the applications was analysed, and the following applications were excluded: entertainment games, delivery shops, applications dedicated to flowers (home green plants), and duplicated results. The final selection included fifty-two applications. The subsequent stage—screening, consisted of excluding applications aimed at identifying types of plants (based on the submitted photo), graphic garden design, food supply (Reason 1) or applications dedicated to large-scale agriculture (Reason 2), and applications older than 5 years (Reason 3). Finally, fourteen unique applications were selected. Their main purpose was closely related to the support growing of house or garden plants (vegetables, fruit, herbs).

Selected mobile applications are presented in Table 1.

**Table 1:** Selected applications.

Application name	Assigned category	Main purpose
Farm Your Yard	House & Home	Supports gardening / vegetable planting
From Seed to Spoon	House & Home	Complete growing guide
Garden Manager	Lifestyle	Garden management
Garden organizer	Lifestyle	Supports growing garden plants
Garden Tags	Communities	Community of gardeners
Gardenize	Lifestyle	Supports monitoring of a garden
Gardroid	Lifestyle	Teaches growing own vegetables
Grow with Jane	Productivity	Guidance throughout the harvest process
Moon & Garden	Lifestyle	Supports growing organic garden
Plant Story	House & Home	Gardening assistant
PlantApp	Education	A guide to growing various plants
Planter	Lifestyle	Garden Planner
Sowing Calendar	Lifestyle	Plant growing guide
Veggie Garden Planner	Home / Lifestyle	Supports planning a vegetable garden

### Data analysis

The raw data includes material from the fourteen applications. Publicly available reviews encompassing neutral, positive, or negative feedback were downloaded and manually analysed as a part of the study. The scale of points awarded by users ranged from 1 to 5. All fourteen applications use the same scale of points (ranging from 1–5) awarded by users. The reviews were based on users’ individual experiences and what they wished to share. Consequently, some of the downloaded opinions contain only brief assessments, such as “Nice up” (Plant Story: 928), “Not bad at all I like it” (Moon & Garden: 9850). Others provide valuable information, expressing not only positive or negative aspects of the applications but also recommendations for their improvement. Thus, all reviews were important to the research process. Approximately 7 980 reviews were analysed using MAXQDA® qualitative coding software. The user reviews are from different years, depending on when the applications became available on the store platform, and the studied period covered the years 2018–2023.

The study applied the methodology proposed by [29], applying recognised content analysis (frequency analysis). In the first step of the procedure, the collected data was reviewed, then initial codes were generated based on emerging thematic issues in individual user feedback and the research questions. In the second step, subcategories for the main thematic areas were selected. In the last step, the frequency of code occurrence in each subcategory was counted. Content analysis based on the identified codes was conducted according to the paradigm of techno-learning ecologies [30]. This paradigm suggests that the techno-ecological environment in which we are immersed extends our learning through cybernetic interactions. The availability of increasingly advanced educational software expands the digital media landscape, enhancing the value of learning outcomes and engagement within the techno-ecological educational ecosystem.

## Results

Despite the common denominator linking the selected applications to support urban gardening, individual applications use different approaches, which is reflected in the assigned categories. The categories indicated in Table 1 are name categories defined in the Google Play Store. The applications also differ in the amount of publicly available feedback. There are applications that offer a small number of available reviews, as well as applications that have a larger number of reviews, nevertheless, all reviews are equally important for gaining an in-depth understanding of the needs, habits, and expectations of the urban gardening application users. The analysis conducted provided answers to the research questions, revealing significant indicators about the applications’ functionalities. The functionalities considered in the “Results” section are presented according to a quantitative criterion, from the functionalities most frequently quoted in the user reviews to the ones that were least frequently mentioned.

### From motivation to engagement

An analysis of motivational factors shows that those searching for an urban gardening application are mostly people who consider themselves as beginners. These mobile applications serve as a guide with tips for individuals who lack horticultural and gardening knowledge. “This app is exactly what I needed! It does everything a newbie gardener needs in order to undertake the planting process for any edible plant” (From Seed to Spoon: 2091). Interestingly, the reasons for taking the decision to start growing food vary. According to some users, their decision to use the application is linked to starting a new activity or hobby. The users who start their adventure with gardening report difficulties in achieving the expected goals. This makes them search for further educational assistance.

Another reason for using the applications mentioned in the reviews, is to receive suggestions or inspiration from friends, family, acquaintances, or colleagues. Users provided examples of how their friends inspired them to start a mini garden, gave specific gardening solutions, such as eco-friendly pots, and recommended a particular mobile application. Users also mentioned finding inspiration to try out these applications from TV shows, radio programs, and social media.

The group of applications’ users includes not only the individuals who have small “gardens” on balconies or terraces but also the ones who have access to a backyard. It was indicated that the decision to use the application was linked with the decision to transform their backyard into a garden for growing fruits, vegetables, or herbs. Another reason given is the opportunity to start gardening activities after moving to a new place of residence. The applications are used to identify plants in the new garden, as well as to document its content.

Another group of users reported a lack of success in their previous gardening experiences. After years of unsuccessful gardening, they decided to find an educational tool that would provide them with correct practices and to guide them through growing plant processes, from planting seeds to harvesting. Another group of users consisted of those who started gardening during a period of social isolation. Their motives were linked to the pandemic. The application not only guided them through the gardening process but also served as a substitute for social interaction.

However, the most common motivation for downloading an application indicated by users is a desire to expand their subject knowledge “The reason I downloaded this app was I saw there was a way to quickly check the companion plants and enemy plants for many different fruits and veggies" (From Seed to Spoon: 1795), “As an amateur gardener it has provided me the much needed support and knowledge required” (Garden Tags: 2061), “From a beginner’s standpoint, I loved this. Seriously it makes the information easy to digest” (Sowing Calendar: 1539).

Not only beginners seek support from gardening applications; experienced gardeners also find them helpful. For these users, the primary motivation for downloading the application was the desire to improve documentation of the progress of their work in the garden.

According to user feedback, garden diaries are usually kept in notebooks. However, this method has the disadvantage of potential loss or damage during gardening. Searching for information written in different parts of the notebook, or on different sheets of paper is also time-consuming. The application simplifies note taking on mobile devices and allows for easy documentation of results through photos. One user noted, “It makes it so much easier than writing it all down on paper. I can log the date sowed, date sprouted, date harvested, and harvest quantity for each variety. Much less effort with this app” (From Seed to Spoon: 2170).

**Fig 2:**
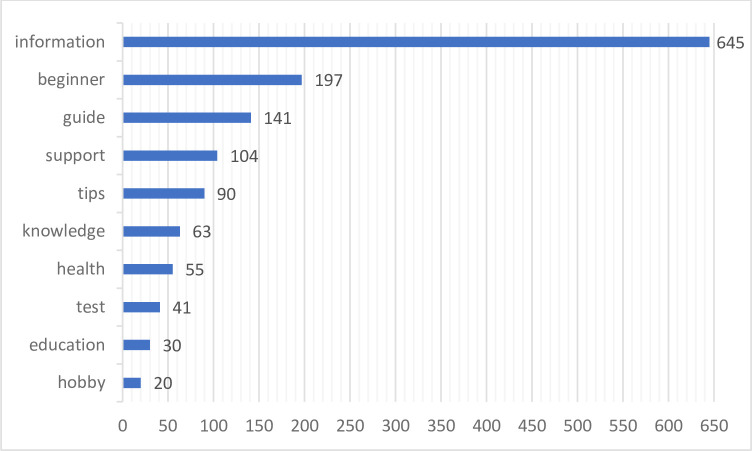
Number of occurrences of a given motive in users’ reviews. Author’s own elaboration.

Users also appreciate receiving notifications from the application about necessary plant treatments or garden care tips, which helps them keep track of their garden. Additionally, the application is also used by people who want to assist others. This is particularly relevant for older generations, including parents and grandparents, who may not be proficient in using the application but still want to utilise its features. Their children or grandchildren often use the application to assist their family. Furthermore, some users have expressed health concerns as a motive for downloading the application. With the guidance provided, individuals can cultivate medicinal plants and learn how to prepare homoeopathic remedies. A summary of the frequency of words describing these motives in user reviews is presented in Fig 2.

### Functionalities facilitating user engagement

#### Source of knowledge

As one of the main motives for downloading the application was the need for knowledge, the quality of the information provided is the most frequently cited reason for respondents’ decision to continue using the app.

The key reason is the range of available information. According to users’ opinions, the application should provide not only basic information on plant cultivation “Very interesting and informative especially for the novice. Impressively detailed” (Moon & Garden: 9728), but also technical data to support the planning and gardening process, e.g., how many plants fit in each square foot/metre, or how much container capacity is needed “It provides a list of herbs, fruit, and vegetables that can grow in your region, helpful information on companion planting, organic pest control, what pests to expect for which plants, recommendations for products to help you grow an organic garden” (From Seed to Spoon: 2300), information related to health issues or even recipes on preparing or processing the harvested fruit and vegetables “This app is great for finding useful info on gardening, managing crops, and even has some recipes (more please! 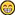)” (From Seed to Spoon: 1217).

Another key motive is the way the application’s content is presented as it may facilitate the use of the application’s resources “This site informative and easy to read and understand” (Sowing Calendar: 1794), “It really helped me with understanding the number plants per square foot and when to plant/transplant into the garden” (From Seed to Spoon: 100), or it may discourage from using the application as it is not adjusted to users’ needs “Might work OK for veteran gardeners who need reminders, but not much help for newbies like me” (Garden Manager: 783). Users also appreciate adequate length of the content provided “There is a range of information available in easy-to-read tables and short pieces of text” (From Seed to Spoon: 462) as well as different channels of communication “Teaches me about planting, growing & harvesting my garden. How and why to plant companion plants, about pests, and so much more. Their videos are great too” (From Seed to Spoon: 837).

#### Reminders

Another frequently desired functionality of the application was a notification system for effective monitoring of gardening progress. Users emphasised that a significant advantage of the app is the elimination of the need to search for information about when to plant, water, prepare soil, or about conducting other maintenance activities. Users frequently declared that they naturally let notifications guide their actions. As one user stated, “I’m a keen gardener but have a lousy memory, this app makes it so much easier to keep track of what I’ve planted and when” (Garden organizer: 1120). The notification system informs users of the optimal times to begin planting particular vegetables, herbs or fruits, taking weather conditions into account and suggesting whether to start planting indoors or outdoors. Users can also set their own notifications. In case of applications that did not have a built-in notification option, users expressed a need for it. One user noted, “Would love to see push notification reminders for planting, watering, harvesting” (Planter—Garden Planner: 2343).

#### Utility

Another essential issue was the quality of the interaction between the application and the user. The reviews indicated that the users expected a simple, straightforward, intuitive use of the app “This is just what I been looking for so easy to use” (Sowing Calendar: 5390). Users pointed out that tasks, for example, the replacement of a previously uploaded plant photo should be done in a single step without having to switch between pages. The application should allow notes to be added quickly and easily, especially to upload pictures documenting plant growth. One user stated, “Easy to add new plants and useful information on the plants on the system” (Planter—Garden Planner: 2939).

User feedback also indicated that attempts to improve the app’s usability could provoke unexpected reactions from users “The previous version was infinitely better, providing an easy-to-read design, with more detailed information & understanding, clearly presented, comfortable to view & absorb the instructions based on the structure & colors of the app design. I will B seeking an app to replace this one. I will B removing this app. Finding it difficult & confusing to understand” (Moon & Garden: 438).

#### Connection to the location

Another useful feature of the application is its connection to the user’s location. The advantages of this functionality include reliability of the weather data provided and the notifications based on it, such as those for watering, impending frosts, “I love the fact that it uses the closest weather station to me to help me plant” (From Seed to Spoon: 928). Users valued customised calendars, suggesting what to plant each month and that would help them keep track of the crops. One user remarked, “Nice, but needs to be adjustable to growing zone, otherwise, the calendars that would be helpful are just confusing” (Veggie Garden Planner: 165). Access to additional site-specific information was also important, such as the best match for the type of vegetables, fruit, herbs planted, the optimal time to start each plant type, and pest problems: “You can choose the zone where you live, and the app will show what veggies you can plant all year round, companions and enemies of the plants, care recommendations, even plant pest and disease!” (Sowing Calendar: 321).

#### Responsiveness

Responsiveness is another key factor in sustaining user engagement. According to users’ feedback, a user expects support from application developers with technical problems. Users appreciate the opportunity to request technical support and receive quick responses, but may also be frustrated by frequent attempts to resolve the issue. This is a major problem for them, especially if there are bugs in the application.

Contact with the service department is crucial for users as it influences further application development and helps to enhance it by adding desired functionalities. Users appreciate that as the garden becomes more diverse, the application also improves. The application’ developers continue to enhance the application and incorporate new features. Sometimes it is in response to user requests. It is noteworthy that users appreciate the quick response, even in situations where it is not necessary, as one of them mentioned: “I sent feedback and within the same hour heard back from Ehsan, who not only answered my request but expanded upon the information made available to us all” (Farm Your Yard: 76). Prompt feedback is highly valued, especially when it is discovered that the problems related to the application performance are not a result of its limitations, but rather due to the parameters or capabilities of the device being used.

#### Sharing options

For many users, the social aspect of the application was essential. These users not only rely on the information provided by the application’s content but also highly value the tips and advice from other friendly people with similar interests on how to become better gardeners. “A hands down brilliant app which connects UK Gardeners, experienced & inexperienced alike, to share gardening tips, in an amazingly friendly & Helpful community. You can share pictures of your garden & plants with people who appreciate it, & be wowed by the array of amazing plants UK gardeners grow” (Garden Tags: 2335).

Some users expressed an interest in combining functionality with social media. One user commented, „Function to Share in Facebook cannot work Fix it please” (Garden Manager: 262). The forum option is often seen as a local feature that enhances effective knowledge sharing. “The ability to share your calendar would be nice as well. A community feature where people can publicly post their calendars & notes on successes & failures would also be helpful. This way users can see what grows well locally” (Farm Your Yard: 122).

#### Additional features

Another critical issue for users is additional application options. They highlighted the benefits of features to add notes and photos for each planted variety and to track plant growth based on uploaded pictures. One user mentioned, “I can add new pictures under each plant like new spikes or new root pictures. So I can keep track of each plant growth stage” (Garden Manager: 1238).

Users also value the connection to online services like YouTube, weather forecast or stores “I love the YouTube video links—they are all short and digestible and makes every gardening endeavor seem achievable even for someone who has only been gardening a couple of years” (From Seed to Spoon: 1854); “I just recently found the option to buy seeds through the app, so exciting!” (From Seed to Spoon: 130).

Another important option is the ability to filter application content according to various criteria such as temperature, indoor, outdoor, companion plants, to specific pests that could harm the plant. Equally important is the choice of diverse ways to display information on planned gardening work, such as a monthly view, a graphical tool to automatically create graphs recording the growth (height and width) of a plant based on log entries.

### Functionalities hindering user engagement

#### Technical issues

In addition to the many positive functionalities presented, users also point out aspects that make the application challenging to use. The most frequent comments relate to technical problems, crashes, or bugs. Using the applications to support one’s own urban cultivation requires hours of entering individual plant data into the app. Users express frustration when the entered data was lost after switching off the application or updating it, “It was 5 stars before the latest update. Now, I cannot adjust any of my plants nor check them off when I thin or harvest” (Farm Your Yard: 141), “today when I opened the app to add some notes on fertilizing plants yesterday, my garden information disappeared right in front of me. One second it was there and the next it was all gone. The screen just says My Garden is Empty. I had so much information with notes and pictures and now it’s all gone. Don’t trust this app!” (original writing, From Seed to Spoon: 582).

Users often criticise the slowness of the applications, especially when adding photos “Really slow when adding a picture or it would just freeze my whole phone” (Grow with Jane: 8239), “the app is so slow. It’s frustrating to use on my phone because the slowness causes unnecessary errors and forces me to redo things that shouldn’t need to be redone” (From Seed to Spoon: 149). Users also point out that the application quickly drained the phone’s battery.

#### Administration issues

Issues related to administration are often associated with a reluctance to set up an account, log in or enter data. For example, one user stated, “there is NO way I’m giving you my first name, last name, birthdate and year and location just to look up plant info” (Garden Tags: 325). Participants also highlight their reluctance to be pressured into purchasing a paid version of the application, receiving unwanted notifications or excessive amounts of advertising, “good app and provides good info but once I saw the free limitation of 3 plants for a garden with an ad-based experience was disappointing” (From Seed to Spoon: 4). However, users agree to watch a certain amount of advertising in exchange for free access to all features of the application, provided the amount is reasonable.

#### Interface

Users present many objections to application interfaces. In addition to issues related to subjective feelings about the colour scheme, banner or logo design, and size of symbols, there was criticism of the screen layout. For instance, one user commented, “Could have better UI (there’s a health section that’s stuck on the top of the page that is useful, but I don’t want to always see it)” (From Seed to Spoon: 1081). It is also important to adjust the application’s display size to fit the phone’s screen well, ensuring the full view without cutting off at the bottom. This allows for full and unobstructed use of all available functions of the application.

#### Missing options

Users also point out several missing features that make the application difficult to use. One of these was the ability to upload their own garden bed layout. Due to the important level of creativity among users and the wide variety of garden shapes, the available layout is often inadequate. Users also express a desire to add plants manually, as many declare that particular species were missing from the application’s resources, “I would like to see a wider variety of plants in the list” (Garden organizer: 12).

Users have indicated that another missing feature is the synchronisation with other devices or applications, such as those that identify plants and insects from a photo. Another feature that is also important to them is the ability to mark completed actions, so they would not have to wonder if, for example, the plant has already been watered. Additionally, users want the ability to edit saved notes. One user commented, “Found I could not edit written text entries very easily once saved” (Grow with Jane: 8125).

Various sorting options would be very convenient, such as “to have more control over the sorting rather than just alphabetical” (Garden Manager: 370) the ability to convert units, as one user mentioned, “An American App. Doesn’t offer metric” (From Seed to Spoon: 2106).

**Fig 3:**
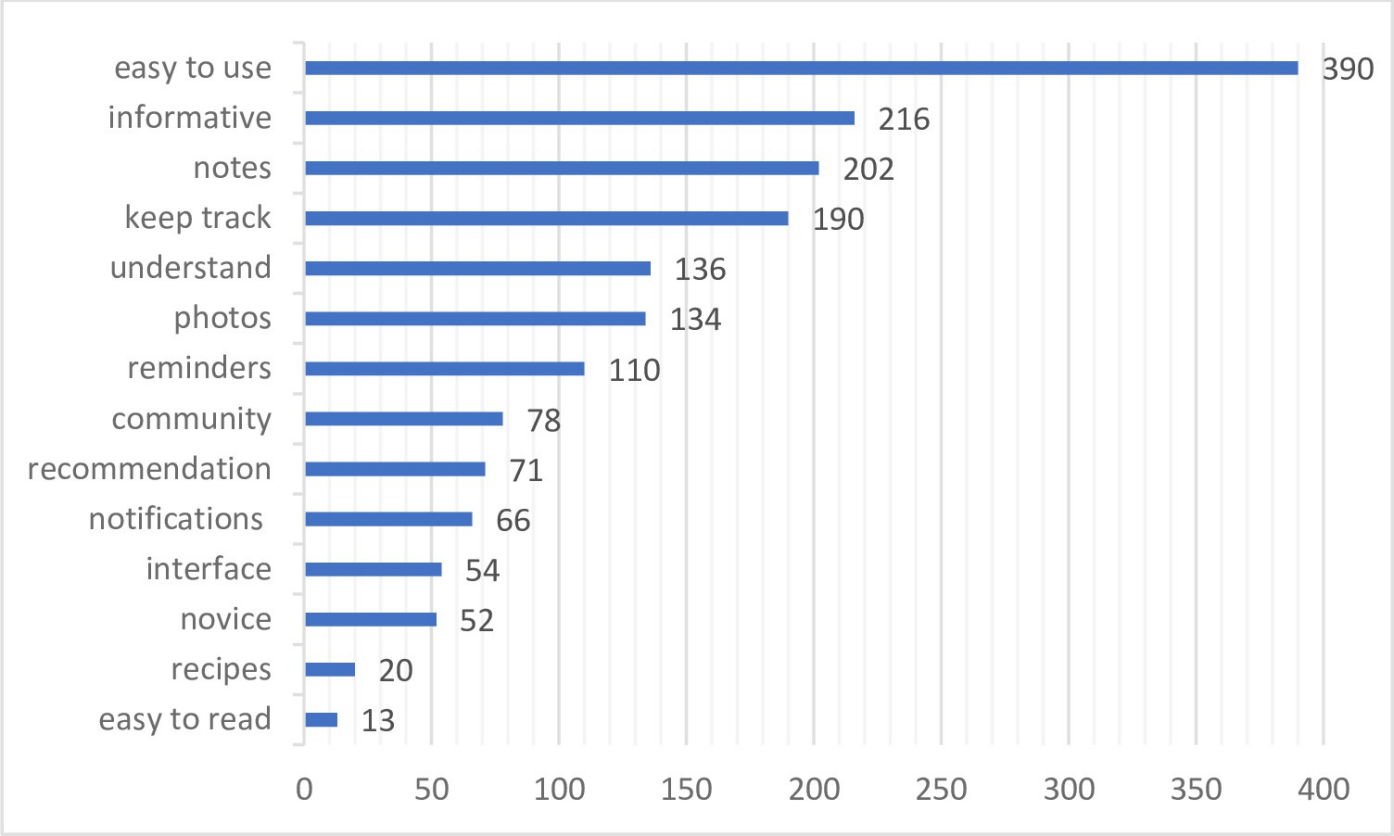
Number of occurrences of a given motive in users’ reviews. Author’s own elaboration.

A summary of the frequency of words describing these features in the users’ reviews is presented in Fig 3.

## Discussion

In addition to their wide-ranging nature the mobile applications offer the possibility of strengthening urban resilience through their use in urban gardening. To fulfil their purpose, they need to be developed in line with the changing needs of a diverse urban community. However, there is still little research dedicated to applications for urban gardening and the needs of their users. Although there is extensive research on applications in fields such as healthcare [31], tourism [32, 33], business [34] or education [35], there is still an underrepresentation of research on small-scale food self-production. An analysis of user feedback -an informal means of digital communication and a digital footprint in a virtual space—from a limited number of available applications of this kind provides an opportunity to determine how current functionalities contribute to strengthening urban residents’ interest in food self-production.

The research indicates that a significant part of the users that use gardening mobile applications are novices. This suggests that these applications should primarily have an educational focus. Developers should therefore make every effort to capture the users’ attention using well-planned and advanced educational resources within the application. At the same time, they should carefully consider the methods used to convey the content. Research indicates that users expect a variety of communication channels tailored to different educational needs, learning styles—visual, auditory, reading and writing, kinaesthetic, as suggested in [36]. Apart from the specialised articles addressed to those who want to extend their knowledge about a specific issue, there is a desire to include quickly accessible and basic references, helpful visualisations and examples or videos. Larger part of the knowledge should be provided in an uncomplicated way–such as map, chart, diagram formats. The information should be detailed—providing exact planting depth, spacing, container size, etc. and accompanied by graphic or photo visualisations. Moreover, users expect to join local social media platforms, viewing knowledge sharing as an important part of the learning process, as other researchers also indicate [37].

To encourage action to slow climate change, the advice given should be based on nature-based solutions. For example, using the natural food chain to deal with aphids or providing friends/enemies companion planting guides, as such advice was of interest to users. The application should include a variety of in-built functionalities to make data easier to understand and process. For instance, information on treatments requiring dosing of products or calculations for germination and sprouting, estimated harvest calculations, and unit conversions, would be well complemented by an automatic calculator.

Subsequent findings indicate that despite the rapid development of information systems and the increasing use of very advanced features by users of smartphones and other mobile devices, easy and intuitive use remains the most desirable aspect of a mobile application’s usability. Comparable results were obtained by [38] when examining the opinions of health applications users. This means that, regardless of the sophistication of the application’s content, the first aspect users assess is its usability, including installation issues, setting up an account, navigating between screens, and accessing resources.

In many of the individual comments from the analysed reviews, both positive and negative, a significant expectation emerged: users want the application to find their location and provide a notification system. Users frequently mention memory problems, so notifications synchronised with current weather conditions and the possibility to tick off completed activities would help make food self-production feel like a hobby rather than a chore. This approach is also recognised in the results of [39], investigating gardening as a recreational activity for urban residents.

The research results also reveal several factors that function as barriers to using the application. These are predominantly technical issues, such as bugs, crashes, slowdowns, freezes, and problems that occur especially after updates, which users nonetheless expect. Additionally, if users do not receive prompt support from the application’s developers to resolve technical problems, they tend to abandon the app. Administrative issues such as the need to create an account, provide personal data, pay for a subscription or deal with intrusive advertising in free applications remain a barrier to downloading or using the application. Finally, the other factors presented in the results section reflect expectations for additional functionality rather than a direct reason for uninstalling the application.

## Conclusions

Mobile gardening applications offer a wide range of possibilities for shaping the pro-environmental behaviour of city dwellers, but their potential is not yet sufficiently recognised and exploited. The study explores the motivations behind a user’s decision to download urban gardening applications and the functionalities that facilitate and hinder their use. These findings are based on available user reviews from fourteen mobile applications. The results indicate that urban gardening applications are primarily educational. Therefore, when planning to create or develop a small-scale urban gardening app, the focus should be on the learning process.

The research conducted was based on an analysis of user reviews available online and did not employ any software for downloading the reviews. As the research is qualitative, its limitation may consist in not representing a broader user group. Therefore, future research should apply quantitative methodology, using the entire review databases, if downloadable. Beside quantitative research, it would be interesting to interview users to know their motivations for urban gardening and how the applications help them with this activity.

The main distinctive feature of the research is that it covers an unexplored and new topic. The analysis of applications for urban food self-production is under-represented in scientific research, even though recently this activity performed by urban residents’ activity should be particularly supported. The present analysis contributes to the literature by exploring factors that are relevant to the development of urban gardening applications. Moreover, given the results obtained, they are relevant not only for applications’ developers, but also for those involved in the urban education process, such as city authorities, urban educators, pro-environmental associations, grassroots activists, who could use mobile applications as a tool in education to support the green transformation of cities.
